# Silicones for Maxillofacial Prostheses and Their Modifications in Service

**DOI:** 10.3390/ma17133297

**Published:** 2024-07-04

**Authors:** Anca Irina Gradinariu, Carmen Racles, Iuliana Stoica, Carmen Gabriela Stelea, Ana-Maria Andreea Simionescu, Alina Elena Jehac, Victor Vlad Costan

**Affiliations:** 1 Department of Oral and Maxillofacial Surgery, “Grigore T. Popa” University of Medicine and Pharmacy, 16 University Street, 700511 Iasi, Romania; 2 Department of Inorganic Polymers, “Petru Poni” Institute of Macromolecular Chemistry, 41A Grigore Ghica Voda Alley, 700487 Iasi, Romania; 3 Physical Chemistry of Polymers Department, “Petru Poni” Institute of Macromolecular Chemistry, 41A Grigore Ghica Voda Alley, 700487 Iasi, Romania

**Keywords:** silicones, maxillofacial prostheses, color, hardness, property modification

## Abstract

The biomedical applications of silicones are countless due to their outstanding properties. In dentistry, silicone for maxillofacial and plastic surgery has become indispensable, from both physiological and aesthetic points of view. In this mini-review, silicone materials for dentistry and facial prostheses are discussed, focusing on their properties and alterations when exposed for long periods to different environments. A significant number of studies reported in the literature have been conducted in vitro, mimicking some of the main degradative factors which have been identified as triggers for discoloration and deterioration of the mechanical properties. Among these, in artificial aging and accelerated natural aging studies, UV radiation is considered the most important. Other weathering factors, biological contamination, and disinfection agents may have dramatic effects as well. Several general properties of silicones are described at the beginning, with a focus on biocompatibility, cross-linking mechanisms, and applications in dentistry and maxillofacial prosthetics. We discuss the ongoing cross-linking and/or possible exudation after manufacturing, which also affects the stability of the prosthesis over time, and possibly the patient. Next, the main environmental factors that affect the prostheses in service are presented, including the role of cigarettes smoke, which has been discussed very little so far. A few aspects, such as biofilm formation, its negative effects, and proposed solutions to overcome this phenomenon regarding silicones, are also described. We conclude by proposing a set of topics for future research and development based on the gaps that have been identified in the literature. Although silicones are probably irreplaceable in maxillofacial prosthetics, improvements in terms of base materials, additives, surface treatments, and maintenance are possible and necessary for long-lasting and safer prostheses.

## 1. Introduction

Anaplastology is a distinct specialty that deals with the design, creation, and manufacturing of various types of prostheses, such as facial prostheses, ocular prostheses, auricular prostheses, and other devices that replace or enhance the appearance and functionality of parts of the body that have been affected by various conditions or traumas. Maxillofacial surgery is a medical and surgical discipline that deals with the diagnosis and treatment of maxillofacial conditions, such as diseases, injuries, and anomalies of the maxilla and face, while dental technology refers to the methods used in the treatment and replacement of teeth and oral tissues. Maxillofacial prosthetics is a branch of anaplastology in close connection with maxillofacial surgery and dental technology, focusing on designing and manufacturing personalized prostheses for patients who have experienced loss of substance or functionality in the face or neck.

The role of maxillofacial prostheses is both functional and aesthetic, and these devices are essential to restoring the quality of life of affected patients. Maxillofacial prosthetic materials have physical properties ranging from hard, stiff alloys and polymers to soft, flexible polymers and elastomers [1]. Among these, silicone rubbers (elastomers) are the most popular, being biocompatible, nontoxic, lightweight, easy to manipulate, and clean, with relatively simple manufacturing (including pigmentation) and mechanical properties that mimic the human tissues and can be adjusted within relatively large limits.

Despite their qualities, the maxillofacial prostheses made of silicones have a relatively short life in service of 7 to 24 months, mainly depending on the method of retention (adhesive or implant), according to a 2010 survey [2]. The causes for prosthesis replacement include color changes, poor maintenance, and silicone tear. In addition to these, other drawbacks of silicone maxillofacial prostheses are a lack of reparability, technique sensitivity, and extrinsic colors peeling/fading [3]. Given the rather high cost of such prostheses, understanding the reasons for these modifications and finding solutions to overcome or to postpone the replacement are crucial.

The factors that have been identified to negatively affect maxillofacial prostheses in service, producing deteriorations in aspect and physical and mechanical properties and leading to patients’ dissatisfaction, are UV radiation, moisture, extreme temperatures, biological fluids, disinfectants and soaps, air pollutants, and mechanical stress. The majority of the reported studies were carried out on specimens prepared from commercial materials which were tested in vitro by exposing them to artificial weathering or accelerated natural aging. The discoloration and deterioration of the mechanical properties were then monitored. Fewer studies concern the role of disinfecting solutions; the effect of biofilm formation on the surface of maxillofacial prostheses; and the roles of air pollutants, cigarette smoke, or beverages. Generally, the reported results have been interpreted with clinical relevance. However, real, worn prostheses are affected cumulatively by all the aforementioned harmful conditions, leading to losses of their properties, including aspect, elasticity, and shape. On the other hand, aside from external factors, not much is known about the long-term properties or stability during wearing of the base materials, which are complex mixtures. Indeed, polydimethylsiloxane (PDMS), which is the main component, shows good weathering behavior [4], good thermal stability, and is hydrophobic, which make it suitable for use outdoors. However, it is permeable for water vapors and several gases, and it is not monodisperse, but contains chains of different end-functionality and macrocyclic compounds. These compounds, as well as many additives that are used in the formulation of the prosthesis material, are not chemically bonded, so they may in theory be extracted from the final device, leading to modifications of the properties. Such additives include pigments, which themselves undergo chemical transformations under UV light, temperature, or moisture, changing the aspect of the prosthesis [5].

All these factors, including physical and chemical interactions, limit the lifespan of the prostheses, and as a result, their study and understanding contribute to the development of improved materials and maintenance routines. It is indeed rather difficult to design and conduct studies on statistically significant numbers of worn prostheses and to take into account all these aspects simultaneously. Without being exhaustive, the aim of this review article is to gather the most relevant information on the stability and modifications of silicone materials for maxillofacial prostheses in service, with a critical perspective. Several general properties of silicones are described, with a focus on biocompatibility, their cross-linking mechanisms, and applications in dentistry and maxillofacial prosthetics. Next, the main environmental factors that affect the prostheses in service are presented, including the role of cigarette smoke, which has been discussed very little so far. A few aspects of biofilm formation, its negative effects, and proposed solutions to overcome this phenomenon with silicones are also presented.

### 1.1. Silicones: Structure, Properties, Biomedical Applications

**Scheme 1:**
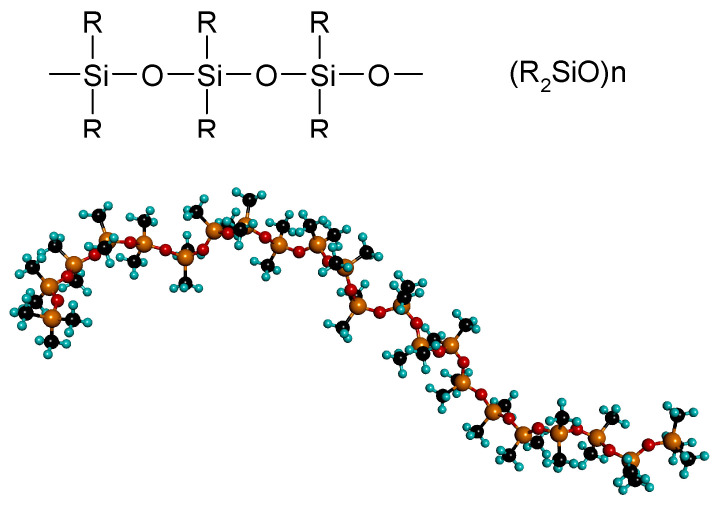
The general formula of polydiorganosiloxanes and the molecular structure of PDMS (Si—orange; O—red; C—black; H—blue).

The generic name “silicones” describes a class of materials based on polydiorganosiloxanes, mostly polydimethylsiloxanes (PDMS), which are generally cross-linked. Polysiloxanes are considered the most important and commercial family of synthetic inorganic polymers [6], and have more than 150 000 different practical applications [7] in various fields, including medical, cosmetics, construction, electronics, energy, and aerospace, to name a few. Polydiorganosiloxanes have structural units with the formula R_2_SiO, R being an aliphatic, aromatic, or functional group, but mostly methyl in commercial silicones (Scheme 1), and this is the reason why the terms “silicone” and “PDMS” are largely used interchangeably.

It has been established that the siloxane bond exhibits distinct particularities in comparison to carbon bonds (Table 1), which impart specific chemical and physical properties to polysiloxanes [8,9,10]. For example, it is characterized by very low rotational energy (close to zero). In the case of polydimethylsiloxanes, the CH_3_ groups freely rotate around the siloxane bond and orient towards the air interface, sheltering the partially ionic character of the Si-O bonds and thus being responsible for the hydrophobic behavior of PDMS. The methyl groups are involved in very weak intermolecular interactions which—besides the high chain flexibility—set the glass transition temperature well below room temperature (typically around −120 °C for PDMS). The unique set of properties which characterizes PDMS also includes very low surface energy, low dielectric constant, good thermal–oxidative and UV stability, extreme resistance to ozone and corona discharges, transparency to visible and UV light, stability towards atomic oxygen and oxygen plasma, film-forming ability, permeability to various gases, and chemical and physiological inertness [11,12].

The biocompatibility of polymers is evaluated by examining their interaction with blood and tissues, depending on their role and the application site. Biocompatibility is the capacity of a material not to induce toxic, trombogenic, allergic, or inflammatory reactions in the blood or tissue. Any response of the host organism is generally unfavorable, except when vascularization is necessary to support living cells [13]. PDMS is recognized as biocompatible and is one of the most tested materials concerning safety [7]. Siloxanes have low general toxicity and no reported genotoxic potential, apart from some data showing inflammatory effects of certain silicone compounds found mainly in cosmetics [14]. There is conclusive evidence indicating that breast implants do not have carcinogenic effects on humans, according to The International Agency for Research on Cancer (IARC) [15]. Various tests have been conducted on animals to evaluate acute oral toxicity, acute skin toxicity, and inhalation toxicity. The results showed a lack of any effect, or very high critical concentrations of PDMS of different viscosities. Also, a non-irritant effect on the skin was observed [12]. Tissue cultures from different warm blood animals did not show deviations from normal growth when they came into contact with liquid, semi-solid, or rubber silicone products [11]. However, the physiological inertia is inherent only for high-molecular-weight polysiloxanes, while low-molecular-weight linear and cyclic volatile compounds may exert negative effects [7]. PDMS is well tolerated in intraperitoneal and intravenous administration [11].

The medical-grade silicones are generally grouped into three categories: non-implantable, short-term implantable, and long-term implantable. Most medical-grade silicones are at least USP Class VI certified. The medical-grade silicone market was worth USD 1.5 billion in 2022 [16], and it continues to grow due to increased demand for medical and cosmetic applications.

The surface and interfacial characteristics are very important for materials in direct contact with biological media. Due to their low cohesive energy and high chain mobility, polydimethylsiloxanes have very low surface tension, varying from 15.7 mN/m in hexamethyldisiloxane to 20–23 mN/m for high-molecular-weight polymers or filled materials [12]. For comparison, the critical surface tension of polyethylene is 31 mN/m, and that of polystyrene is 36 mN/m. For most medical applications, the hydrophobic character of PDMS, its ability to allow oxygen diffusion and to withstand sterilization and disinfection conditions, and its biostability [13] are key properties. However, in other situations, the hydrophobicity of silicones is considered a drawback, and modifying their surface properties can limit the risks. For example, microbial infections may be favored when silicones are in contact with host tissue [17], and various solutions to solve this problem have been proposed.

Given their physiological inertness, excellent compatibility with blood (low interaction with plasma proteins) [18], very low toxicity, and anti-adhesive properties, silicone elastomers have been largely used for medical devices, such as blood pumps; heart stimulators**;** mammary prostheses; draining implants in glaucoma; artificial skin; maxillofacial reconstruction; esophagus replacement; contact lenses; catheters; medical adhesives; lubricants for needles, syringes, catheters, etc.; seals and gaskets; scar treatment sheets; respiratory masks and denture relining [6,13,17,19]; and for covering shape memory alloys for artificial muscles and implants [20]. They exhibit reduced tissue reactions in comparison to similar non-silicone implants, along with gradual covering by epithelial cells. Compared to other hydrophobic polymers, PDMS is very permeable to the diffusion of different substances, including gases, water vapors, and drugs, which makes them suitable for transdermal and drug-delivery applications [21]. For example, the permeability to oxygen of silicone rubber is 25-fold higher than that of natural rubber [22]. The water vapor permeability of silicone rubber is very high, comparable to collagen, and threefold higher than for polystyrene [22].

### 1.2. Silicones in Dentistry and Maxillofacial Prostheses

Being a liquid at room temperature, PDMS has to be cross-linked for applications as self-standing material or device in maxillofacial prostheses. The cross-linked silicones are soft elastomers with mechanical properties that can be adjusted according to what is needed by different cross-linking mechanisms, or by compounding with various fillers. Besides rigorous hygiene during their preparation and purity of the base silicones, the mechanism, catalytic system, and curing conditions are essential in silicones for medical applications.

**Scheme 2:**
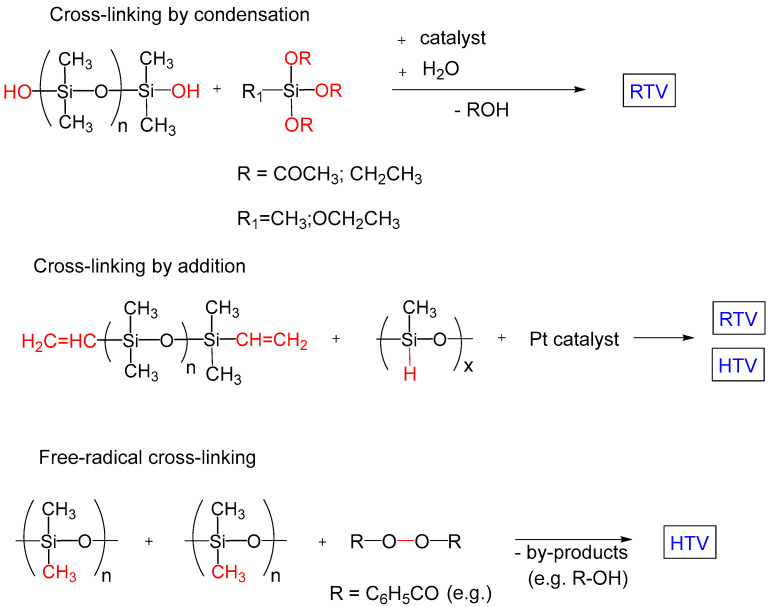
The main cross-linking mechanisms in maxillofacial silicones.

There are several types of silicone materials for prostheses, classified according to their cross-linking conditions as “room temperature vulcanizing” (RTV) and “high temperature vulcanizing” (HTV), each class containing one-component or two-component basic silicones and specific curing mechanisms, i.e., condensation or addition in RTV silicones and free-radical or addition mechanisms in HTV silicones, respectively. The main types, their advantages and drawbacks, and most known brands were reviewed multiple times [23,24,25,26,27,28]. The reagents for each type of reaction are depicted in Scheme 2.

PDMS with –OH or vinyl chain ends and relatively high molecular weights are used as the main components. Poly(methylhydrogen)siloxane oligomers are added in small proportions (usually 10%) as cross-linking agents in addition to cross-linking, while vinyl groups can be attached in very small amounts to the polysiloxane chain to increase the vulcanization rate and cross-linking density in free-radical systems. The cross-linking by condensation has the advantage of low energy consumption, being conducted at room temperature in the presence of atmospheric humidity, but the reaction generates by-products and uses catalysts (tin-based, for example), which may be harmful. The by-products (e.g., acetic acid or ethanol) may have side effects, like shrinkage of the prosthesis or skin irritation. The addition system does not produce any by-products, which guarantees excellent dimensional stability. The process can be carried out either at ambient temperature or at a high temperature. On the other hand, it uses catalysts based on platinum (or other noble metals), which are very sensitive to impurities. The addition process is characterized by a short working time, selective adhesion, and low compatibility with extrinsic pigments [24]. The free radical cross-linking uses peroxides as initiators, needs a high temperature for vulcanization, may involve certain risks, and generates by-products. These by-products create pores in the cured silicone (which is avoidable by using high pressure) and can lead to depolymerization at higher temperatures.

**Figure 1:**
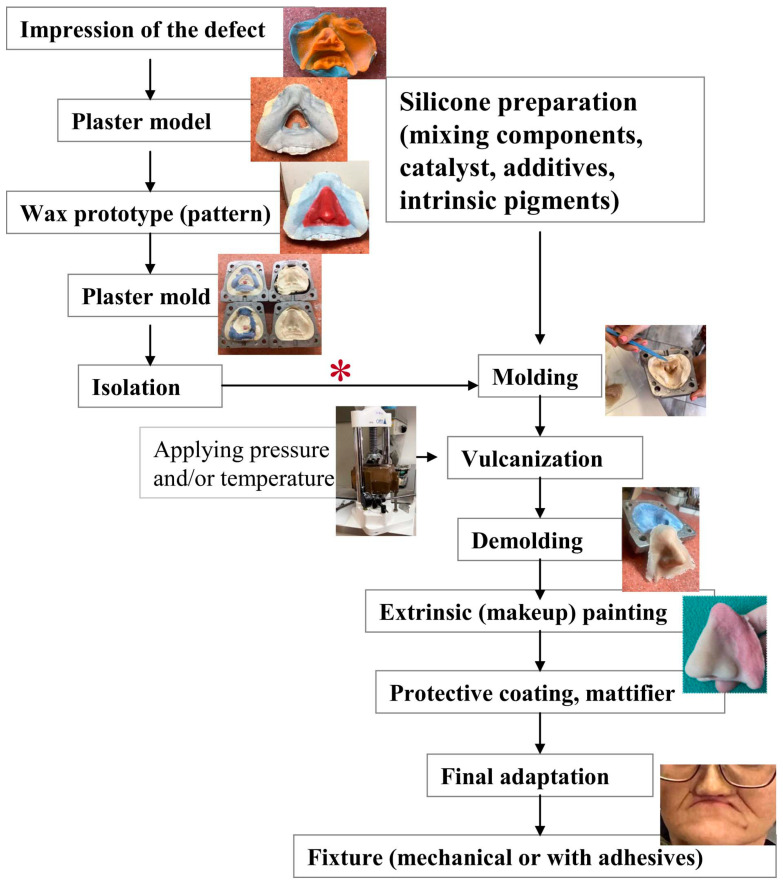
Manufacturing process of a maxillofacial prosthesis (* supplementary steps: in certain situations, an acrylic base is fabricated, a primer is applied, then the silicone mixture is poured).

For prosthesis fabrication, the RTV silicones have the advantage of using low-cost molds made from plaster and dental stone (gypsum). However, the condensation system is unsuitable for thick sections due to the limited ability of moisture to permeate deeply [24]. On the other hand, the peroxide HTV system yields silicones with good mechanical and thermal properties, but poorer aesthetic properties. Addition-type silicones, either RTV or HTV, are the most used in maxillofacial prostheses at this moment. Each producer provides variable compositions, containing base silicones and catalysts and including fillers and optional additives (usually silica, opacifiers, intrinsic pigments, thixotropic agents). The processing of silicones for facial prostheses is dictated by the cross-linking mechanism, the properties of the base silicones (molecular weight, viscosity, functionality, additives, etc.), and the final use. The manufacturing techniques of silicones for these kinds of applications are still largely based on handcrafting (Figure 1).

In addition to the aforementioned types, UV-curable silicones, foaming silicones, and silicone block copolymers are newer options that are less commonly employed [26,28], while various modifications to the structure, composition, fillers, etc., have been the subject of intensive research [23,29,30]. Up-to-date techniques like 3D printing are gaining increasing interest [24]. There are indirect and direct 3D-printing approaches. The former implies fabrication of rigid polymer molds for silicone cross-linking, while the latter refers to layer-by-layer deposition and curing of silicone materials. Either classical RTV or photoinitiated curable silicones are used. Although there are commercial formulations for the 3D printing of silicones, this is still a growing field of research, including block-copolymers and blends [31].

**Figure 2:**
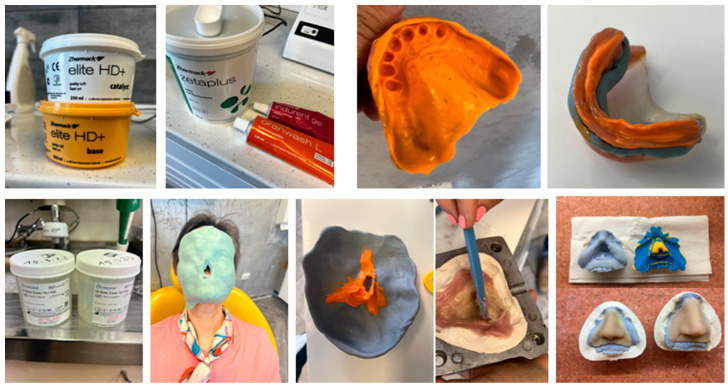
Silicone materials for impression used in dentistry and handcrafted facial prostheses.

Dental and maxillofacial fields are interconnected and the silicone materials used in these applications are similar (Figure 2), especially in the particular case of soft lining. Silicones are used in dentistry as impression materials, in prostheses, and in adhesives [32,33,34]. Among the characteristics of silicones, some are essential for these kinds of applications: the biological inertness, the compatibility with human tissue and body fluids, the rheological behavior ensuring accurate replication of teeth and gum lines, the skin-like consistency, the odorless and tasteless characteristics, the ability to withstand sterilization and to resist bacteria, the absence of corrosive or staining effects, the shape retention, and the durability. In addition, they are easily processed by casting, as they are fluid and have good elastic memory and volumetric stability, and they are easily colored close to the gum tissue. Dentists use silicone-enhanced impression molds for bridge and crown reconstruction, as well as in false gum prosthetic models. Their properties make silicones a good choice for the accurate replication of teeth and gums, with very good approximation of tissue consistency, optimal cushioning and comfort, shape retention, and durability. These are short-term applications; thus, alterations to the silicone’s properties are less probable.

There are also cases of complete or partial edentation, where soft relining and resilient materials are needed. Soft liners are used for reducing local pressures [35,36], acting as a cushion to attenuate the masticatory forces. Silicones are considered the best choice for long-term use as soft liners compared with plasticized acrylics. For example, although storage in artificial saliva leads to an increase in hardness [37] and significantly affects the color integrity of silicone materials [38], their color stability is better than that of acrylic resin materials. This is because their higher hydrophobicity reduces the sorption of water-based solutions. On the other hand, their hydrophobic behavior leads to poor lubrication, which produces patient discomfort; thus, the use of silicone soft liners in contact with saliva is still limited to 1 year [39]. Solutions may be found to overcome this drawback, one example being the use of non-surface treated silica as filler, which increases the water adsorption [40]. Addition RTV-type silicones are preferred as soft liners [38], being superior to condensation silicones [34], but HTV (peroxide, free radical type) silicone composites have also been proposed for this application [41]. Since the long-term applications of silicone materials in dentistry are limited, their stability during wearing has been less investigated.

Maxillofacial prostheses address defects in any part of the head or neck, including the ears, nose, and adjacent tissue, and are essential to restoring aesthetics and function to increase a patient’s quality of life. Due to its outstanding physical, biological, and chemical properties, including skin-like flexibility, intrinsic transparency, light weight, processability, and aesthetics, PDMS has been the most widely used maxillofacial prosthetic material since the 1960s. Other materials commonly used for the fabrication of facial prostheses are acrylic resins, acrylic copolymers, vinyl polymers, and polyurethane elastomers, but silicone is the closest to an ideal prosthetic material [42]. Silicone elastomers can form the main body of the prosthesis alone or in combination with other materials, or can serve as lining materials [43].

Facial prostheses need to look as natural as possible, and since the mechanical properties of silicone materials mimic the human tissue, together with appropriate pigments and manufacturing techniques, they ensure the best results. Thus, medical-grade silicones are used in the majority of external (ex vivo) facial prostheses as the main material [24]. The most common maxillofacial silicones on the market are MDX4-4210, A2186, A-223, M-511, A-2000, A-2006, Q7-4635, Q7-4650, Q7-4735, SE-4524 U, S-6508, 382, and 399, under brands like Silastic, Factor II, Cosmesil, Nusil, Prestige, etc. Five commonly used maxillofacial materials were analyzed by Aziz et al. [40] for tear strength, tensile strength, percentage elongation, hardness, water absorption, and water contact angles. The study concluded that none of the tested brands was ideal, but Factor II showed superior properties (tear strength, softness, and ease of manipulation). The mechanical properties of the most used materials were compared by Hatamleh et al. [1], and newer products like MPDS-MF, MED-4920, SY-28, ZY-1, and TechSil S25 were brought into consideration from this perspective.

In the particular field of dentistry and prostheses, the supports most often used for silicone parts are acrylic resins. One drawback of silicones in general is their incompatibility with almost any other material. This makes the bonding of soft liners with the denture base, as well as silicone prosthesis with fixtures, challenging. In these situations, solvent-based primers [44] or trifunctional silane coupling agents [45] are used. On the market, there are dedicated primers and formulations for each type of silicone base. The primer is practically a compatibilizing agent, reacting with both materials via hydrogen bonds and covalent bonds [46]. Nevertheless, separation of silicone from acrylic base in maxillofacial prostheses is a significant reason for replacement, representing around 12% of causes [2].

## 2. Modifications of Silicone Maxillofacial Prostheses in Service

### 2.1. Factors Affecting the Long-Term Use

During wearing, maxillofacial prostheses are exposed to several harmful factors, like UV radiation, moisture, extreme temperatures, biological fluids, disinfectants and soaps, biological contamination, air pollutants, mechanical stress, and chemical interactions, leading to the loss of their properties, including aspect, elasticity, or even shape. These physical and chemical changes limit the lifespans of the prostheses; thus, their study and understanding help in the design of improved materials and maintenance routines.

In the case of silicones, the main chemical process that affects the service life of the prostheses is the continued cross-linking, which can seriously modify the mechanical characteristics [24]. When the cross-linking reaction is not completed during the manufacturing process, the reaction continues at a slower rate long after the prosthesis is finished, leading to hardening; increased elastic modulus, glass transition temperature, and viscoelasticity; and decreased maximum stress, strain, and tear strength [47]. The color may change as well as a result of additional cross-linking and side reactions involving impurities or the catalyst [48]. Although it is very important, the completeness of the cross-linking is often overlooked.

**Figure 3:**
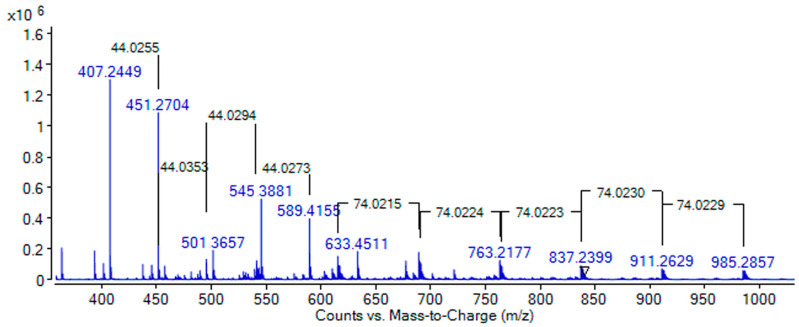
ESI-MS spectrum, in positive ionization mode, of an extract from a cross-linked maxillofacial silicone (M511) [49]. The peaks were identified as siloxane oligomers, for example: the peak at *m*/*z* = 407.2449 corresponds to [D_5_ + 2H_2_O + H^+^], where D_5_ is decamethylcyclopentasiloxane; the mass difference of 44.02 corresponds to [Si-O]; and 74.02 corresponds to [(CH_3_)_2_Si-O] unit.

On the other hand, when the starting materials contain compounds and additives that are not chemically bonded, there is a risk of such compounds being expelled, either naturally or during cleansing. Consequently, this has an impact on the characteristics of the prosthesis and even on the patient. For example, low-molecular-weight compounds of siloxane nature have been extracted in THF from a silicone for maxillofacial prostheses after curing (Figure 3) [49], showing that the cross-linking was not complete and/or non-cross-linkable compounds were present in the starting materials. However, this aspect has not been sufficiently investigated, and more data are needed to establish whether or not the unbonded compounds in the prosthesis may separate during wearing and/or negatively affect the patient.

Weathering, especially UV radiation inducing photo-oxidation processes, seriously affects the mechanical properties of silicone materials and produces color alterations. Simulated skin secretions have also been found to affect the elasticity and hardness of silicones [50]. Microbial growth adversely affects both mechanical properties and the appearance of the prosthesis, causing skin irritation and infection [51], while cleaning products and disinfectants may also degrade silicones [52], as will be shown in the next paragraphs. In the meanwhile, inherent color changes might appear in time as a result of changes to the physical and mechanical properties caused by internal factors, or during vulcanization. A darkroom study [53] showed that the molding-stone color and vulcanization temperature both affect the degree of color change, which exceeds the perceptible thresholds.

**Figure 4:**
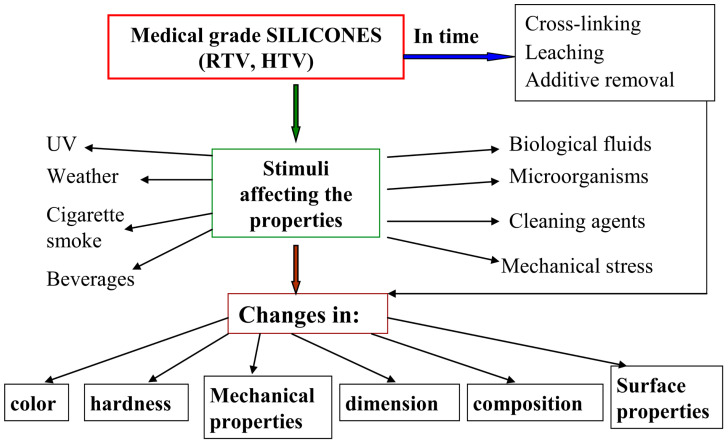
The main factors identified to affect the long-term properties of silicone maxillofacial prostheses.

The main aspects concerning the long-term stability of maxillofacial silicones are summarized in Figure 4.

### 2.2. The Effects of Environmental Degradation

Silicones are considered as being resistant against the effects of weathering [4]. However, maxillofacial prostheses have been observed to exhibit serious modifications in time, resulting in a restricted average lifespan of approximately 2 years. The main reason for replacement is discoloration and mismatch with the surrounding tissues, which diminishes the patient’s satisfaction. Besides gradual discoloration of prostheses in a service environment, degradation of the physical, static, and dynamic mechanical properties of the silicone have been reported [54]. The degradation process occurs due to ultraviolet radiation, pollution, and variations in temperature and in humidity [55]. Most of the reported studies focused on accelerated natural daylight aging or artificial daylight aging.

The photo-oxidative degradation is described as a three-step reaction, involving initiation, propagation, and termination [46]. During the initiation process, free radicals are generated and specifically cleave the methyl side groups in PDMS rather than the Si-O bonds, which have high energy (Table 1). The formed silyl radicals react with oxygen in the propagation step to produce oxy-, peroxy-, and secondary polymer radicals, resulting in chain scissions [46,47]. Termination by recombination creates new cross-links. Thus, there is continuous competition between chain scission and inter-chain bonding. Additionally, volatile degradative compounds may also be generated. The main structural modifications are in the molecular weight distribution [47]. As a result, the mechanical properties suffer modifications during weathering; either hardening or softening may occur depending on the balance between these reactions [56], and other material’s physical properties may be affected. The alterations to the mechanical properties under natural daylight aging, accelerated artificial daylight aging, human body perspiration and sweat, and cleaning solutions were reviewed [1]. It was concluded that, although the reports were not always consistent, changes in the mechanical properties of silicone elastomers for maxillofacial prostheses do occur and depend on the silicone type, brand, and the pigments or other additives used, as well as on the testing conditions.

It has been established and it is generally accepted that the ideal hardness of maxillofacial prostheses should be 25–35 Shore-A indentation units, similar to that of the missing facial tissue [57]. Eleni et al. investigated the mechanical behavior of non-pigmented facial prosthetic addition-curing silicones (Elastomer 42, TechSil S25, and Cosmesil M511) exposed to outdoor weathering for 1 year [47]. The elasticity parameters and hardness exhibited different evolutions in the first two materials, with an increase in hardness versus the third silicone, which became softer. Thus, although all these commercial elastomers were of the same type and were processed similarly, small differences in their compositions triggered different degradation mechanisms (i.e., either secondary cross-linking or chain scission predominated).

Three RTV (A-2000, A-2006, and A-103) and one HTV (M-511) silicone (Factor II) were subjected to outdoor weathering for 6 months in an area of tropical Southeast Asia, a climate with high humidity and ultraviolet radiation, and their surface roughness (SR) and mechanical properties were investigated [56]. The SR, tensile strength, and elongation were adversely affected, but the changes differed between the silicone types. A-103 was found to be the most stable in the tested conditions, followed by A-2000.

The mechanical properties of maxillofacial silicone elastomer MED-4210 (Factor II), which was reinforced with different proportions of TiO_2_ nanoparticles, were measured after applying different extra-oral aging methods, i.e., dry storage in the dark for 6 months, storage in simulated sebum solution for 6 months, storage in simulated acidic perspiration for 6 months, accelerated artificial daylight weathering for 360 h, storage in antimicrobial silicone-cleaning solution for 30 h, and mixed aging in sebum under UV light for 360 h [58]. It was observed that TiO_2_ nanoparticles had an anti-aging role, improving the mechanical properties of the silicone elastomer. However, significant changes in the mechanical properties of the silicone samples with or without TiO_2_ nanoparticles (that is, increases in all studied parameters) were reported after immersion in simulated sebum solution. The result was attributed to decomposition of the siloxane bonds in the presence of fatty acids. A similar effect was found for samples stored in simulated acidic perspiration due to the catalytic effect of the acidic medium. The TechSil-S25 silicone elastomer was tested in the same way [59], and it was found that the Shore-A hardness increased after immersion in acidic solution and exposure to accelerated light aging, but decreased significantly after immersion in simulated sebum solution or combined exposure to sebum and light.

Artificial weathering has been reported to cause significant color changes in prosthetic silicone elastomers. One of the spectrophotometric methods used to assess these changes (CIEL*a*b*) calculates the color variation between pristine and exposed samples, ΔE* (Equation (1)), and values higher than 3 characterize clinically unacceptable modifications [60].

ΔE* = [Δ(L*)^2^ + Δ(a*)^2^ + Δ(b*)^2^]^1/2^, (1)

where L*, a*, and b* are the CIELAB coordinates (L*—lightness, a*—red-green axis, b*—yellow-blue axis); ΔL* = L_1_* − L_2_*; Δa* = a_1_* − a_2_*; and Δb* = b_1_* − b_2_*.

The pigments used in maxillofacial elastomers and their testing methods according to ASTM, e.g., for assessing the lightfastness (“the ability of a material to withstand color change on exposure to light”), were reviewed by Gary and Smith [5]. Given the fact that silicones are transparent to UV radiation and highly permeable to moisture and many gases, the sensitive pigments incorporated or applied on the surface, are exposed and may degrade. During exposure to UV radiation, the organic pigments especially suffer chemical transformations which are reflected in discolorations. Thus, although the presence of pigments is critical for ensuring a color match with the surrounding tissues, the pigments used have a significant influence on the color stability of the prosthetic materials. The data on the color stability of maxillofacial silicone prostheses are not consistent in the specialized literature due to the very large number of pigments of different natures that are available and the different testing conditions. Generally, ceramic pigments are recognized as ensuring greater color stability, while intrinsic organic pigments or external makeup pigments are more prone to chromatic alterations [24,60]. Oil-based pigments combined with opacifiers and certain silicone pigments have been found to protect maxillofacial elastomers, but yellow silicone pigment significantly affects the color stability [61]. Meanwhile, it has been found that unpigmented and pigmented specimens alike experience color changes in different testing conditions [61,62]. The color stability can be improved by using additives like barium titanate, titanium dioxide nanoparticles, and opacifiers [24,48,63]. TiO_2_ is the most widely used due to being inexpensive and readily available. Varying amounts can be recommended depending on the predominant shade (under 5% for yellow pigment and 5–15% for red or blue shades) [64]. A review by Gupta et al. [48] on the color stability of maxillofacial prosthetic materials concluded that “an ideal maxillofacial silicone exhibiting good color stability in various human and environmental aging conditions is yet to be identified”.

Differently pigmented RTV Episil silicone elastomers for maxillofacial prostheses were tested for color changes after exposure to the same radiant energy. The color changes, measured spectrophotometrically, were found to depend on the irradiation time and initial color of the samples [54]. Some samples were more stable, with color changes not detectable by the eye, while others showed significant color changes which were detectable by the eye, but still clinically acceptable.

Silastic MDX4-4210 silicone, unpigmented and intrinsically pigmented with cosmetic or ceramic pigments, was exposed to accelerated aging, simulating the deterioration caused by rainwater, dew, and exposure to sunlight UV energy (UVB) (direct and indirect sun energy), and then to disinfection with effervescent tablets or neutral soap [60]. The spectrophotometric measurements showed color alterations, with the type of pigment and the disinfection method used having important influence. The ΔE values increased with the aging time, as expected. The ceramic inorganic pigment ensured better preservation of the color, while the makeup led to higher levels of discoloration, presumably assigned to higher particle sizes.

Thixotropic agents have been added to the silicone mixtures to prevent development of porosity after polymerization. The impact of different amounts of thixotropic agent on the color degradation of three elastomers (A-2000, A-2006, and A-2186) combined with pigments was investigated during artificial aging [65]. It was observed that the amount of thixotropic agent had a more significant impact on color stability than the type of pigment used in the silicone elastomers. A-2006 exhibited the highest color stability, while A-2186 was the most affected by the increased amount of thixotropic agent.

The effect of natural outdoor weathering has also been investigated [56,62,66]. Outdoor weathering provides a more accurate representation of the natural environment, allowing for observations of how prostheses are modified in real-life situations [48]. However, most studies have been conducted by placing the samples inside cabinets protected by glass, which makes it impossible to measure the impact of factors such as dust or air pollutants. It is rather difficult to compare data due to variations in solar radiation, temperature, humidity, dampness, air pollutants, etc., besides differences in the brand and composition of the investigated samples and in testing methods. Local weather conditions influence the observed color changes, and it seems that humidity and rainfall have a greater effect on colored elastomers than heat and sun [61]. Comparing the best-known silicone elastomers and distinguishing between pigmented and nonpigmented homologues might provide an overview; however, it may not establish a definitive hierarchy of the materials.

A comparative evaluation of the effect of outdoor weathering on the color stability of A-103 and A-2000 silicones for maxillofacial prosthesis was reported [66]. The A-103 RTV silicone group with pigmentation and the nonpigmented A-2000 RTV silicone group showed the maximum changes in color after six months of environmental conditioning. In the tested environment, pigmented A-2000 showed better color stability than A-103 RTV silicone. Visually perceptible and clinically unacceptable color changes occurred for the A-103, A-103 pigmented, and A-2000 elastomers (ΔE > 3), whereas only the A-2000 pigmented elastomers exhibited clinically acceptable changes (ΔE < 3) in that study.

The RTV silicone elastomer A2186 (Factor II), unpigmented or pigmented with natural inorganic dry-earth pigment or synthesized organic pigments, was tested in different weathering conditions [62]. Discoloration was observed irrespective of the pigmentation; thus, it was presumed that the elastomer itself made a contribution to color change. However, without regard to the L* component of the CIE color system, the lightfastness (ΔE* ab) of the nonpigmented elastomer fell within ASTM category I (excellent lightfastness). The pigmented materials behaved differently depending on the pigments used. The study concluded that the tested materials were in ASTM category II (very good lightfastness), without providing a predictable color change. Two pigments (burnt sienna and alizarin red) gave the most unexpected results when blended into the elastomer, and a wide variation in color was observed depending on the location.

Farah et al. found that nonpigmented samples suffered less color alteration in natural outdoor weathering than in artificial aging conditions [61], but the changes were significant and were attributed to an increase in cross-linking in the elastomer network upon exposure to UV light. The induced structural changes were considered responsible for modifications in the transmission and scattering of light, thus leading to color changes. The same study reported that pigmented M511 specimens demonstrated good color stability, with a maximum ΔE of 2, below the acceptability threshold when stored in darkness or exposed to outdoor weathering.

### 2.3. Exposure to Cigarette Smoke and Beverages

Although acknowledged as a factor producing staining and discoloration to maxillofacial prostheses [5,62], the effect of cigarette smoke on silicone elastomers has been investigated very little. Based on clinical observations, deterioration of silicone maxillofacial prostheses is severely accentuated in smoking patients.

Cigarette smoke contains over 4000 compounds [67], such as carbon monoxide, formaldehyde, radioactive polonium, ammonia, nickel, arsenic, tar, and heavy metals such as lead and cadmium [68]. Many of these compounds are harmful and might be adsorbed in silicones. Besides the fact that smoking is a public health problem, according to the World Health Organization, the effect of cigarette smoke on maxillofacial prostheses should be regarded as complex. Therefore, not only should the unpleasant discoloration of the prostheses be a focus, but the alteration of other properties, as well as the adsorption and persistence of undesired compounds, should also be considered. At the present, the phenomenon has not been systematically studied, and there is a gap in the literature concerning the stability of maxillofacial prostheses exposed to cigarettes smoke, but patients are advised to avoid smoking based on visual observations in practice.

The effect of cigarette smoke on the color stability of Silastic 44210 was investigated in a “smoking chamber” more than 40 years ago [69]. The samples exposed to the smoke (120 cigarettes in total) were surface-cleaned prior to spectrophotometric analyses. A slight increase in the dominant wavelength and a large increase in color saturation were reported. The authors also proposed a solvent extraction step which was able to restore the original color parameters after staining with smoke.

More recently, maxillofacial silicone M511, both pigmented with various tones and un-colored, was investigated for color changes after exposure to cigarette smoke [70], and the efficiency of hand soap and chlorhexidine gluconate mouthwash in terms of removing the stains was evaluated. The study revealed significant color changes in all tested specimens exposed to smoke, over the acceptable threshold.

**Figure 5:**
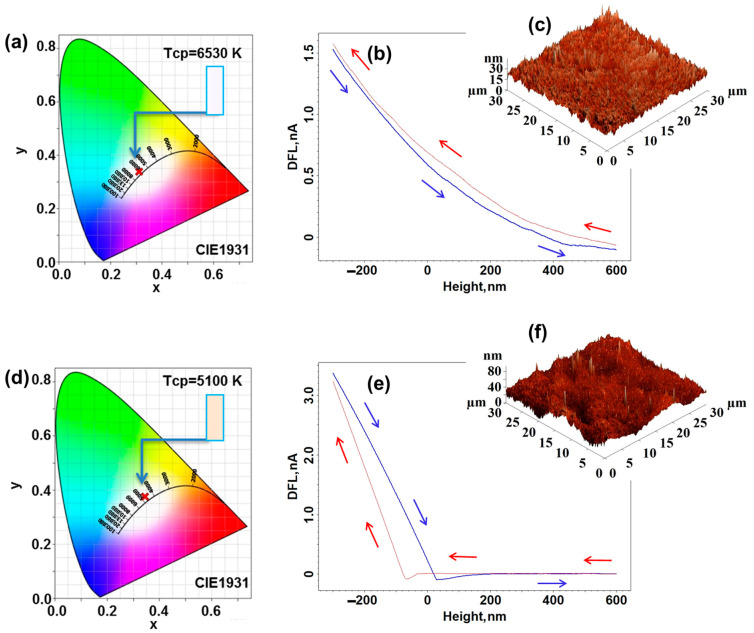
CIE 1931 (x, y) chromaticity diagrams (**a**,**d**) the red x sign indicates the temperature of the colour, representative force–distance curves (**b**,**e**) the red arrows indicate the trajectory of the approach red curves and the blue arrows indicate the trajectory of the retract blue curves, and three-dimensional height AFM images (**c**,**f**) collected for an unpigmented sample before (**a**–**c**) and after being subjected to the smoking process (**d**–**f**) [49].

Our investigations [49] showed more complex implications of cigarette smoke on the maxillofacial silicone M511. Important chromatic and optical changes were measured on a thin, unpigmented sample exposed to 60 cigarettes (rinsed with water prior to analysis): the dominant wavelength increased from 494 nm in the pristine sample to 564 nm, while the recorded illuminance decreased considerably, showing approximately a 23% decrease in transparency. The color temperature also decreased, clearly showing a pronounced yellow tint (Figure 5a,d). Other properties were also affected: the contact angle decreased, the roughness (by AFM) increased (Figure 5c,f), and the local mechanical properties changed (Figure 5b,e). Structural changes in the silicone material were observed based on FT-IR and thermal analyses, consistent with the hypothesis of secondary cross-linking. The observations were in line with other studies reporting increases in the glass transition temperature and modifications to the thermal stability of Silastic MDX4-4210 upon exposure to UV radiation [71]. The presence and persistence of lead in the sample exposed to cigarette smoke after extraction with solvents was evidenced, and several adsorbed organic compounds were identified. It was also observed that THF does not completely remove the stain, but extracts many compounds, some of siloxane nature, which are present in the base material (Figure 3). More studies will be necessary in order to further validate the reported data on a larger number of samples and on different silicone materials.

Insufficient research has been conducted on the impact of colored or acidic beverages on the long-term aspect and properties of maxillofacial silicones. Only recently did Chugh et al. report a study on the color changes of Silfy, A-2186-F, and VST-50 materials immersed in tea or coffee at drinking temperature [72]. The authors observed color changes for all of the silicones exposed to the beverages, with a significantly higher ΔE for A-2186-F and a greater effect of coffee compared to tea.

### 2.4. Effect of Cleaning Agents on Maxillofacial Prostheses

Cleaning and disinfecting are necessary and required in order to avoid microbial growth and skin irritation. This routine can extend the lives of prostheses, but on the other hand, cleansing and disinfecting solutions, such as sodium hypochlorite solution, commercial disinfectants, chlorhexidine gluconate, soaps, and effervescent tablets, might deteriorate the maxillofacial silicone.

The effect of cleaning solutions, either alone or in combination with aging conditions, was investigated, focusing on alterations to the color and mechanical properties [58,59,60]. For example, disinfection with conventional and plant extract solutions combined with artificial aging was tested on MDX4-4210 maxillofacial silicone associated with pigments and an opacifier (ZnO) [63]. A slight, clinically acceptable increase in the hardness was found, while the color changes were significant, generally clinically unacceptable regardless of the cleaning solutions used.

A disinfectant solution should be optimally effective at reducing the microbial biofilm without harming the silicone prosthesis or supporting parts. During cleaning and disinfecting, digital friction is to be avoided, since it may promote microcracks or the detachment of pigments on the surface. When neutral soap was used to clean the prostheses, generally with short and gentle friction, important modifications were reported: higher ΔE values [60] and significantly reduced hardness [63,73].

The combined effect of pigment type, disinfection solutions, and artificial aging on the A-2000 (Factor II) silicone elastomer was studied [73]. Chlorohexidine 0.2% was found to be the most suitable agent for disinfection of the prostheses, while sodium hypochlorite had a color-fading effect on the silicone specimens. The samples containing brown pigment and those disinfected with neutral soap showed the greatest changes in color (ΔE 7.7 ± 1.3) after artificial aging.

A medical-grade silicone was tested for hardness and color stability after the application of different disinfection methods which simulated 1 year of clinical service, i.e., microwave exposure and immersion in sodium hypochlorite, neutral soap, and disinfecting soap [52]. The hardness decreased irrespective of the method used, while color changes reflected in ΔE values of 1.51–4.15 were found. It was concluded that microwave exposure is a suitable method for the disinfection of silicone, while sodium hypochlorite solution is not recommended.

Silastic MDX4-4210 silicone materials with different compositions (no opacifier, or containing BaSO_4_ or TiO_2_) were subjected to a combination of disinfecting with effervescent tablets, neutral soap, or chlorhexidine gluconate and accelerated aging [74]. A progressive increase in hardness was observed after using cleansing products, especially when an opacifier was present. The modifications were within the acceptable clinical range, and were assigned to “continuous polymerization of the silicone” (probably referring to ongoing of cross-linking) in the presence of disinfecting agents and artificial aging.

When discussing the long-term modifications, apart from color and hardness changes, the dimensional stability is very important. The roles of water, soap water, alcohol, and herbal disinfectant on the dimensional stability of RTV silicones were evaluated upon immersion in the respective disinfectants for 45 days [75]. Significant shrinkage was observed, with maximum modifications with the use of normal water, followed by soap water and alcohol-based disinfectants, while herbal disinfectant resulted in the fewest dimensional changes. The reasons for these modifications might have been the removal of various compounds remaining in the material after cross-linking, like by-products, catalysts, plasticizers, or low-molecular-weight silicones.

### 2.5. Attachment of Microorganisms and Methods to Prevent It

The formation of biofilms due to fungal, bacterial, and mixed infections is a very common problem encountered in implants and external prostheses, irrespective of the material employed. However, the adhesion of bacteria is significantly influenced by surface characteristics. A biofilm represents a multicellular community of bacteria attached to solid surfaces, causing infections, since bacteria in biofilms are 1000 times more resistant to antibiotic treatment than planktonic microorganisms [76,77]. In the particular case of silicones, the low surface tension and, therefore, high hydrophobicity are primary reasons why PDMS is prone to protein adsorption and bacterial adhesion [78,79]. Nevertheless, since microorganisms have different surface properties (e.g., *Escherichia coli* have both hydrophobic and hydrophilic regions in the outer membrane layer), their adhesion to silicones depends on specific interactions in each case, mainly on the level of hydrophobic interaction between the cell and the polymer surface. A large variety of microorganisms can be found on silicone medical devices, resulting in damage and sometimes in infection [51,80].

Biofilms on silicone rubber voice prostheses are the major cause of failure and replacement of these devices [81]. Both bacterial strains and yeast are involved, and their adhesion is interactive: adhering bacteria can suppress or stimulate the adhesion of yeast mediated by saliva. *Rothia dentocariosa* and *Staphylococcus aureus* were identified in biofilms on explanted prostheses, while *Candida albicans* is one of the yeasts generally held responsible for silicone rubber deterioration.

Surface topography plays a very important role in biological processes, such as cell attachment, motility, proliferation, differentiation, and regulation of gene expression [79]. The porosities or microcracks of silicone elastomers, either inherent or produced during manipulation and wear, make them more vulnerable to microbial adhesion [51,82,83]. For example, it has been reported that the implants’ topography and microstructure have great importance in bacterial attachment [84,85], and the biofilm formation tendency is different on the concave and convex sides of flexible devices [83]. Indeed, a non-cross-linked PDMS, such as silicone oil, coated on polypropylene plastic surfaces of an automatic urinometer, irrespective of viscosity, was found to significantly inhibit biofilm formation by Gram-negative and Gram-positive bacteria, as well as fungi such as *C. albicans* [86]. Similarly, when soft liners used in dentistry and maxillofacial prostheses were evaluated in contact with saliva and nasal secretions [87], low adhesion of *Candida albicans* was reported on heat-polymerized silicone soft liner (Molloplast B) with a smooth surface.

Different strategies have been proposed to reduce the biofilm formation on silicones, especially aiming at surface modifications, preferably without using antimycotics or antibiotics due to the risk of inducing resistant strains. Increasing surface hydrophilicity is often considered as the best solution to overcome this phenomenon. Surface modification includes corona, plasma and laser treatments, etching, surface oxidation, hydrolysis, grafting of hydrophilic monomers and polymerization, coating, and direct chemical bonding [79]. Metal (gold, titanium, silver, colloidal palladium/tin) coatings, plasma surface treatment, or surface chemical modifications (e.g., with quaternary ammonium silane or perfluoro-alkylsiloxane) have been reported to achieve superior performance depending on the microbial strains and silicone device. Other strategies have been reviewed [79,80], including the use of surface-active molecules (biosurfactants) or bulk modification by blending, copolymerization, interpenetrating polymer networks, and functionalization.

Anti-adhesive polymers can be used for this purpose, for example, zwitterions like 2-methacryloyloxylethyl phosphorylcholine (MPC) [76]. It has been reported that covalent grafting of nontoxic polymers (containing EGdPEA and DEGMA) that resist bacterial attachment, mediated by thiolene addition on the silicone surface, may reduce the bacterial coverage by 99% [88]. Chemical grafting of poly-acrylic acid onto silicone rubber films using gamma irradiation as a means to increase hydrophilicity, followed by immobilization of ZnO nanoparticle as an anti-biofilm, was found to be effective in the inhibition of bacterial adherence [77]. It has been demonstrated that polysiloxanes grafted with N-acetyl-L-cysteine (NAC) can eradicate mature bacterial biofilms [89,90,91], since NAC is a strong biofilm inhibitor in spite of being a weaker antibacterial compound [90], and is subjected to enzymatic degradation [92]. It was inferred that its mechanism of action is based on increased surface wettability, resulting in reduced bacterial adherence to substrates [93]. Covalent bonding of NAC to silicones has proven to be an effective solution, provided that the modification degree is relatively high [91].

The inner surfaces of facial prostheses are in contact with soft tissues and body fluids, facilitating colonization by microorganisms and the formation of biofilms. Rigorous cleaning or use of inappropriate cleaning agents can lead to damage to the silicone rubber, which increases the incidence of biofilms [51]. Multispecies biofilms have been detected on silicone facial prostheses, including in margin areas that are not directly adjacent to implants [94]. Moreover, discoloration of facial prostheses has been described as being fungal-driven. For example, a fungus of the genus Penicillium was isolated from affected areas of worn nasal prostheses exhibiting black discoloration [95]. In another study on worn prostheses made of pigmented silicone elastomer VST-50HD (Factor II) [51], significantly increased microbial colonization was found on prosthesis-covered skin compared to healthy skin, and *Candida* spp. was exclusively isolated from prosthesis-covered skin and from prostheses. It has been suggested that standard cleaning regimes do not remove all the microbial cells from the surface of the prosthesis, and microorganisms remain embedded in the defects.

Several compounds have been reported to be effective in protecting maxillofacial silicones from microbiological contamination. TiO_2_ nanoparticles incorporated into maxillofacial silicone A-2006 (Factor II) have shown antimicrobial effects against Staphylococcus aureus, Escherichia coli, and *Candida albicans* biofilms [95]. The addition of clotrimazole to in vitro silicone samples was shown to be effective in inhibiting fungal growth, while nystatin was found to be ineffective, and this outcome was ascribed to the instability of nystatin in the acidic medium generated during silicone cross-linking [86].

Various disinfectants (chlorhexidine gluconate, sodium hypochlorite, neutral soap, white vinegar, and effervescent tablets) had different efficacies on *S. aureus*, *E. coli*, and *C. albicans* biofilms formed on maxillofacial silicone [95]. Most of these were effective against *E. coli* biofilm formation, while 100% vinegar solution and effervescent tablets tended to be the least effective disinfectants on *C. albicans* and *S. aureus* biofilms.

## 3. Conclusions and Perspectives

In this mini-review, we gathered the most important aspects concerning the long-term stability of maxillofacial silicones, along with examples that we considered to be relevant. Otherwise, numerous studies can be found on natural or simulated aging of maxillofacial silicones, focusing on UV and moisture as the main triggers and following the same aspects: color changes assessed spectrophotometrically and hardness changes, sometimes accompanied by investigation of other mechanical properties.

However, based on the literature survey, we identified several aspects that remain to be more thoroughly studied, as follows.

- Besides UV radiation and weathering, comparatively little information is available on cigarette smoke, air pollutants, or other factors that might affect the long-term use of these prostheses.
- Most studies were conducted on model samples, while reports on real cases of worn prostheses are scarce. Thus, the combined effect of the complex set of factors affecting the lifespan of such devices is poorly known.
- Materials considered safe based on appropriate standards are used in maxillofacial prostheses according to the manufacturers’ indications, but their long-term side effects are generally not verified in common practice. However, alterations to mechanical properties over time or exudation of certain components might occur, which would affect the lifespan of the definitive prosthesis and might be harmful to the user.
- Modified materials with better resistance to the growth of microorganisms are needed on the market.
- New materials with improved resistance to weathering and wearing should be compared with the commercially available ones. Such investigations are missing.

More studies on the stability, degradability, and property modification during the use of silicone maxillofacial prostheses are necessary. There is always room for improvement in terms of materials, additives, surface treatments, and maintenance for long lasting and safer prostheses.

## Figures and Tables

**Table 1:** The characteristics of the siloxane bond in comparison with carbon bonds (adapted from [8]).

	Si-O-Si	C-C-C	C-O-C
Bond length, Å	1.63	1.54	1.42
Bond angle, degrees	130	112	111
Bond energy, kcal/mol	106	83	86
Bond barrier, kcal/mol	0.2	3.6	2.7
Rotational energy, kcal/mol	0.78	3.3	-

## Data Availability

No new data were created or analyzed in this study.
